# Changes in the Gene Expression Profiles of the Hypopharyngeal Gland of Worker Honeybees in Association with Worker Behavior and Hormonal Factors

**DOI:** 10.1371/journal.pone.0130206

**Published:** 2015-06-17

**Authors:** Takayuki Ueno, Hideaki Takeuchi, Kiyoshi Kawasaki, Takeo Kubo

**Affiliations:** 1 Department of Biological Sciences, Graduate School of Sciences, The University of Tokyo, Bunkyo-ku, Tokyo, 113–0033, Japan; 2 Faculty of Pharmaceutical Sciences, Doshisha Women's College, Kyotanabe, Kyoto, 610–0395, Japan; University of North Carolina, Greensboro, UNITED STATES

## Abstract

The hypopharyngeal glands (HPGs) of worker honeybees undergo physiological changes along with the age-dependent role change from nursing to foraging: nurse bee HPGs secrete mainly major royal jelly proteins, whereas forager HPGs secrete mainly α-glucosidase III, which converts the sucrose in the nectar into glucose and fructose. We previously identified two other genes, *Apis mellifera buffy *(*Ambuffy*) and *Apis mellifera matrix metalloproteinase 1 *(*AmMMP1*), with enriched expression in nurse bee and forager HPGs, respectively. In the present study, to clarify the molecular mechanisms that coordinate HPG physiology with worker behavior, we first analyzed whether *Ambuffy*, *AmMMP1*, *mrjp2 *(a gene encoding one of major royal jelly protein isoforms), and *Hbg3* (a gene encoding α-glucosidase III) expression, is associated with worker behavior in 'single-cohort colonies' where workers of almost the same age perform different tasks. Expression of these genes correlated with the worker’s role, while controlling for age, indicating their regulation associated with the worker’s behavior. Associated gene expression suggested the possible involvement of some hormonal factors in its regulation. We therefore examined the relationship between ecdysone- and juvenile hormone (JH)-signaling, and the expression profiles of these ‘indicator’ genes (nurse bee HPG-selective genes: *mrjp2* and *Ambuffy*, and forager HPG-selective genes: *Hbg3 *and *AmMMP1*). Expression of both ecdysone-regulated genes (*ecdysone receptor*, *mushroom body large type Kenyon cell specific protein-1*, and *E74*) and JH-regulated genes (*Methoprene tolerant* and *Krüppel homolog 1*) was higher in the forager HPGs than in the nurse bee HPGs, suggesting the possible roles of ecdysone- and JH-regulated genes in worker HPGs. Furthermore, 20-hydroxyecdysone-treatment repressed both nurse bee- and forager-selective gene expression, whereas methoprene-treatment enhanced the expression of forager-selective genes and repressed nurse bee-selective genes in the HPGs. Our findings suggest that both ecdysone- and JH-signaling cooperatively regulate the physiological state of the HPGs in association with the worker’s behavior.

## Introduction

Social insects have highly organized societies that are often viewed as superorganisms [1]. In these societies, individuals effectively divide their tasks, such as reproduction, nursing, foraging, and guarding the colony, to maintain colony activity (division of labor). The physiology of each individual is appropriate to the task they perform. Elucidation of the mechanisms underlying the regulation of each individual’s physiological state will clarify how the division of labor is established in social insects. In most social insects, including honeybees, bumblebees, ants, and termites, the physiological state of each individual is associated with the task they perform. Furthermore, in most of these cases, the task shift and physiological change occur in association with caste differentiation [2–5]. In contrast, the age-polyethism of the worker honeybee is unique in that task shift and physiological change occur in a single caste [6].

In the European honeybee, *Apis mellifera* L., the roles of the workers (labor caste) change depending on their age after eclosion [6]. The lifespan of a worker is usually 30 to 40 days, from spring to autumn; young workers (generally, less than 13 days after eclosion) take care of the brood in the hive by secreting royal jelly (nurse bees), whereas older workers (more than 15 days) collect nectar and pollen outside the hive (foragers) [6–8]. In association with this age-dependent role change of workers, various physiological changes occur in many tissues/organs, such as the brain, fat bodies, pheromone-producing gland, and hypopharyngeal glands (HPGs) [9–16]. Among them, the physiological changes that occur in the HPGs, a paired exocrine gland in the worker’s head, is quite intriguing, as they appear to correlate directly with the workers’ tasks. The HPGs undergo structural and functional/physiological changes. In nurse bees, the HPGs are well developed and mainly synthesize major royal jelly proteins [12, 14], whereas, in foragers, the HPGs shrink and mainly synthesize carbohydrate-metabolizing enzymes that process nectar into honey, such as α-glucosidase III, α-amylase, and glucose oxidase [12, 14–16]. The HPG physiology in workers is plastic and is modulated by the colony demand, as well as the worker’s role. For example, in colonies in which the brood is decreased in number, older workers tend to retain well-developed HPGs like nurse bees [17]. In queenless colonies, where no newly emerging workers are supplied and thus older workers need to take care of their brood, older workers continue to synthesize major royal jelly proteins in the HPGs and work as nurse bees [18]. These preceding findings clearly indicate that the physiology of the HPGs reflects the behavior of workers.

To analyze the molecular mechanisms underlying the coordinated regulation of the HPG physiology and worker behavior, we previously searched for genes whose expression in the HPGs differs between nurse bees and foragers in normal colonies as candidates that regulate expression of genes of *mrjps* which encode major royal jelly proteins and/or *Hbg3*, a gene encoding α-glucosidase III, or other ‘indicator’ genes of HPG physiology. We identified a gene encoding a *buffy* homolog (*Ambuffy*) whose expression was higher in nurse bee HPGs than in forager HPGs, and a gene encoding a *matrix metalloproteinase 1* (*MMP1*) homolog (*AmMMP1*) whose expression was higher in forager HPGs than in nurse bee HPGs [19]. *Ambuffy* and *AmMMP1* are thought to be involved in intracellular signal transduction and extracellular matrix degradation in the HPGs, respectively [19]. Expression of these genes in the HPGs can also be ‘indicators’ of the behavioral state of workers, because these genes are differentially expressed in the HPGs: both *mrjp2* and *Ambuffy* are expressed in nurse bee HPGs, whereas *Hbg3* and *AmMMP1* are expressed in forager HPGs. The molecular mechanisms underlying the regulation that coordinates the expression of genes related to HPG physiology with worker behavior, however, remain to be clarified.

In normal colonies, structural and functional changes of the HPGs are associated with the age-related role changes in worker honeybees [6, 12, 14–16]. Whether *Ambuffy* and *AmMMP1* as well as *mrjp2* and *Hbg3* are regulated in association with the worker’s behavior or age, however, is unknown, because task transition usually proceeds along with aging of the workers in normal colonies. Earlier studies using a ‘single-cohort colony’, in which worker honeybees of almost the same age were obliged to perform different tasks, indicated that task is typically a better physiological predictor than age of the workers [20, 21]. In the present study, to clarify the mechanism by which the physiological state (expression of these ‘indicator’ genes) of HPGs changes in conjunction with worker behavior, we first analyzed the expression of *Ambuffy* and *AmMMP1* as well as *mrjp2* and *Hbg3* in the HPGs of nurse bees and precocious foragers, which perform foraging activities earlier than usual despite being almost the same age as nurse bees, derived from single-cohort colonies.

Based on previous studies and this single-cohort study, there are forager- and nurse-biased genes regardless of age. Our findings also imply that some endocrine factors might coordinately regulate the physiological state of the HPGs and worker behavior. Preceding studies indicated that hormonal factors coordinately regulate individual physiology and worker behavior. Juvenile hormone (JH) is suggested to be involved in the regulation of changes in the physiological state of various organs/tissues including the brain, fat bodies and some exocrine glands, such as HPGs and pheromone-producing glands [9, 11, 13, 22–24]. In particular, the relationship between physiological state of the HPGs, as well as fat bodies and JH has been well studied [13, 22–24]. As for the HPGs, the JH titer in the hemolymph increases with behavioral development, and application of JH increases the enzymatic activity of α-glucosidase in the HPGs and contracts the gland tissues [13, 22]. Although these evidences clearly indicate that JH plays a central role in the coordinated regulation of HPG physiology and worker behavior, the molecular mechanisms responsible for the JH-dependent changes, including the induction of forager HPG-specific genes such as *Hbg3*, remain to be elucidated, due to lack of knowledge on the molecular action of JH in the honeybee. On the other hand, recent studies suggest that nutritional state of workers is involved in regulating ovary physiology and vitellogenin titer in the hemolymph, both of which influence behavioral development of workers via ecdysone signaling [10, 25–30]. In addition, our previous studies indicated that some genes encoding ecdysone signaling molecules are preferentially expressed in the mushroom bodies, a higher center of the insect brain [31–33], suggesting possible role of ecdysone signaling in the regulation of worker honeybee behaviors [31–35]. However, there are only few reports of the possible involvement of ecdysone, including ecdysone-signaling molecules, in the regulation of role-dependent HPG physiology.

In the present study, we evaluated the possible role of JH and ecdysone in worker task transition. We investigated the relation between the endocrine systems (ecdysone and JH signaling) and gene expression profiles of the HPGs. Our findings suggest that ecdysone signaling and JH signaling are activated in forager HPGs, and ecdysone signaling represses the expression of both nurse bee- and forager-selective genes, while JH signaling upregulates forager-selective genes and downregulates nurse bee-selective genes.

## Results

### Quantification of *mrjp2* and *Hbg3* transcripts in the HPGs of nurse bees and precocious foragers derived from single-cohort colonies

**Fig 1:**
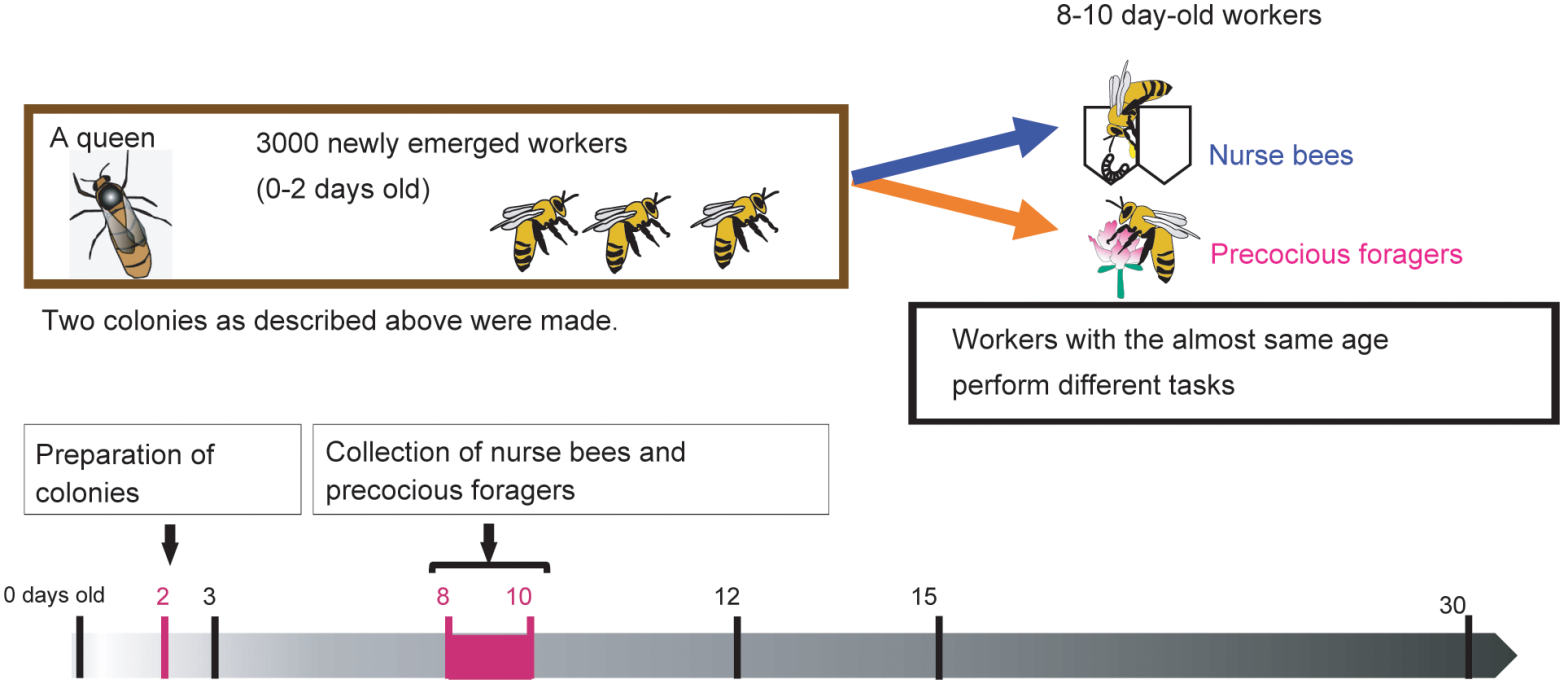
Scheme for preparation of single-cohort colonies. The horizontal arrow indicates the course of workers’ lifetime in single-cohort colonies. Numbers with vertical lines indicate the age of workers. Two colonies, each initially consisting a queen and approximately 3000, 0–2 day old workers were establish to induce division of labor independent of worker age. Six to eight days after establishment, 8–10 day old workers which performed either nursing or foraging were collected. The latter workers were defined as ‘precocious foragers’.

To examine whether *mrjp2* and *Hbg3* as well as *Ambuffy* and *AmMMP1* are regulated depending on the worker’s behavior, we quantified the transcripts of *mrjp2*, *Hbg3*, *Ambuffy*, and *AmMMP1*, in the HPGs of worker honeybees derived from ‘single-cohort colonies’, in which workers of almost the same age are obliged to engage in different roles. The single-cohort colony initially comprises young workers of almost the same age (0–2 days-old), and in the absence of old workers some young workers initiate foraging earlier than usual whereas other workers are engaged in nursing the brood (Fig 1) [20]. The workers that initiate foraging earlier than usual are called precocious foragers. We expected that we could determine whether *Ambuffy* and *AmMMP1* expression correlate with the worker’s role or with its age by comparing gene expression in the HPGs of nurse bees and precocious foragers derived from the same single-cohort colonies.

**Fig 2:**
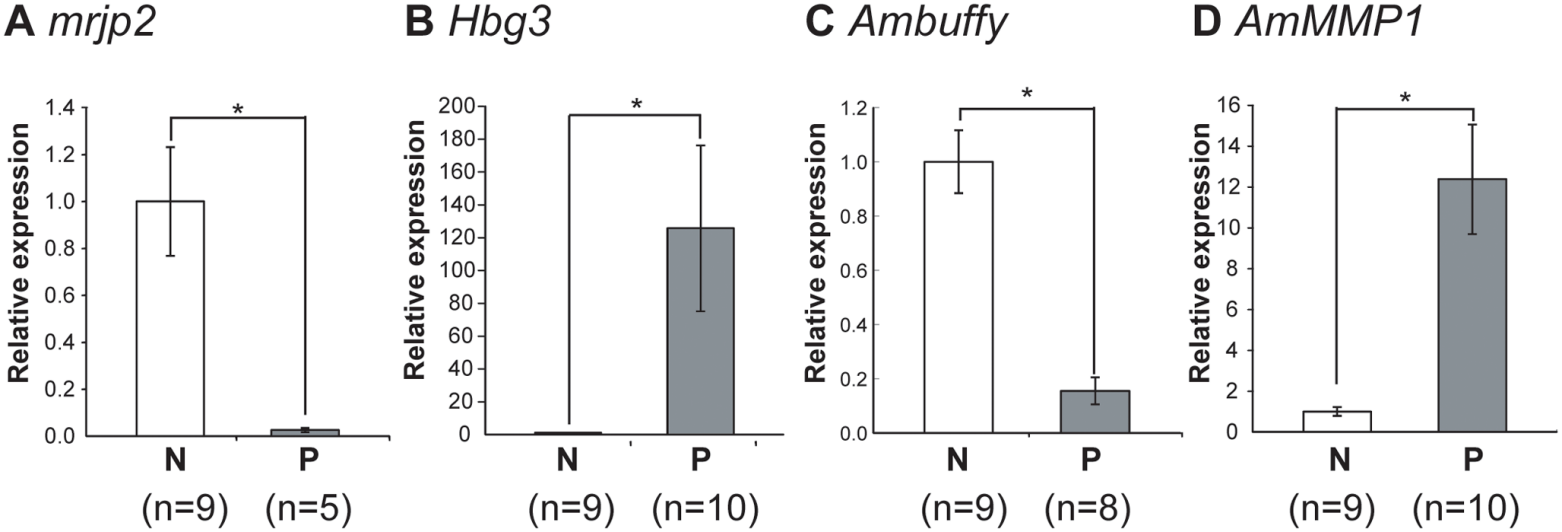
Quantification of gene transcripts in the HPGs of workers derived from ‘single-cohort colonies’. Nurse bees (N) and precocious foragers (P) were collected from two single-cohort colonies. Relative mRNA levels of *mrjp2* (A), *Hbg3* (B), *Ambuffy* (C), and *AmMMP1* (D) in the HPGs of workers derived from two single-cohort colonies are indicated with standard error. The amount of mRNA in the HPG of nurse bees is defined as 1. Transcript amounts were normalized with that of *ribosomal protein 49* (*rp49*). Asterisks indicate significant differences between nurse bees and precocious foragers (*, p < 0.05; t-test). The expressions of *mrjp2* and *Ambuffy* in some workers could not be quantified because signal intensities of these samples were lower than the detection threshold. Thus, number of samples are different from each gene.

First, we examined whether expression of *mrjp2* and *Hbg3* correlates with the worker’s role or age using a single-cohort colony. Comparison of the mean expression levels in pooled samples from two colonies revealed that *mrjp2* expression was approximately 40-fold higher in nurse bees than in precocious foragers (Welch’s t-test, p<0.01), whereas *Hbg3* expression was approximately 130-fold higher in precocious foragers than in nurse bees (Welch’s t-test, p<0.05) (Fig 2). These expression patterns of *mrjp2* and *Hbg3* were similar to those of nurse bees and foragers derived from normal colonies (Fig 2) [19].

### Quantification of *Ambuffy* and *AmMMP1* transcripts in the HPGs of nurse bees and precocious foragers derived from single-cohort colonies

We then quantified the *Ambuffy* and *AmMMP1* transcripts in the HPGs of nurse bees and precocious foragers derived from two single-cohort colonies. Comparison of mean expression levels in pooled samples from two colonies revealed that the *Ambuffy* expression level was approximately 6.0-fold higher in nurse bee HPGs than in precocious forager HPGs (Welch’s t-test, p<0.0001), whereas the *AmMMP1* expression level was approximately 13.0-fold higher in precocious forager HPGs than in nurse bee HPGs (Welch’s t-test, p<0.01) (Fig 2). These expression patterns of *Ambuffy* and *AmMMP1* in the HPGs of nurse bees and precocious foragers derived from single-cohort colonies were similar to those in the HPGs of nurse bees and foragers derived from normal colonies [19].

These findings indicate that, like *mrjp2* and *Hbg3* expression, expression of *Ambuffy* and *AmMMP1* closely correlates with the worker’s role, while controlling for age. The fact that the gene expression profiles of the HPGs are linked to the worker’s behavioral role, even in single-cohort colonies, suggests that some endocrine systems cooperatively regulate HPG physiology and honeybee worker behavior. In addition, the drastic changes in gene expression levels of *Hbg3* and *AmMMP1* between cohorts suggests that these ‘indicator genes’ of the forager HPGs are very sensitive to the worker’s task transition.

### Expression analysis of genes related to ecdysone signaling in the HPGs of workers associated with the role change

Findings from the gene expression analysis using the single-cohort colonies suggested that regulation of the expression of ‘indicator genes’ was associated with the worker’s behavior. Therefore, we next evaluated the molecular machinery, including ecdysone- and JH-regulated genes, which seem to govern the expression of the ‘indicator’ genes in HPGs. To investigate whether the function of ecdysone signaling in the HPGs changes in association with the role change of worker honeybees, we quantified the amount of *ecdysone receptor* (*EcR*), *E74*, and *mushroom body large type Kenyon cell specific protein-1* (*Mblk-1*) transcripts in the HPGs of nurse bees and foragers derived from normal colonies.

*EcR*, *E74*, and *Mblk-1/E93* are well-characterized key genes in the ecdysone-signaling pathway. Previous studies from other groups indicated that, during *Drosophila* metamorphosis, ecdysone induces the expression of these genes and the gene products function as transcription factors, leading to the induction of genes related to morphogenesis [36, 37]. Until recently, almost nothing had been known about the involvement of ecdysone signaling in worker behavioral differentiation in any tissue, because ecdysone titer in the hemolymph undergoes very little change throughout workers’ life, although a small peak is observed in 3-day old workers [38]. We previously demonstrated, however, that some ecdysone-regulated genes, including *EcR*, *E74*, and *Mblk-1*, are expressed preferentially in the mushroom bodies (a higher center) of the adult honeybee brain, suggesting the possible roles of these genes in brain function as well as the possible regulation of brain function by ecdysone-signaling in adult honeybees [31–33]. Furthermore, the physiological state of the ovary (ovarian mass) influences the behavioral development of workers, and expression of ecdysone-regulated genes in the ovary is correlated with ovary size [28]. In addition to these previous studies, our experiments using single-cohort colonies suggested the involvement of some hormonal factors in coordinated regulation of the physiological state of the HPGs and worker behavior. Therefore, we evaluated whether ecdysone signaling is involved in the regulation of gene expression profiles in the HPGs in a behavior-dependent manner, although the direction of the expression of these genes in the HPGs between nurse bees and foragers could not be predicted.

For this experiment, 9 to 14 nurse bees and foragers were collected at the same time from a single colony, and gene expression was compared using a total of four batches of HPG samples derived from workers from four different colonies. We used pooled samples collected from normal colonies to minimize the effect of individual variation in gene expressions. The nurse bees and foragers were collected based on their behaviors as well as HPG development, as described previously [12].

**Fig 3:**
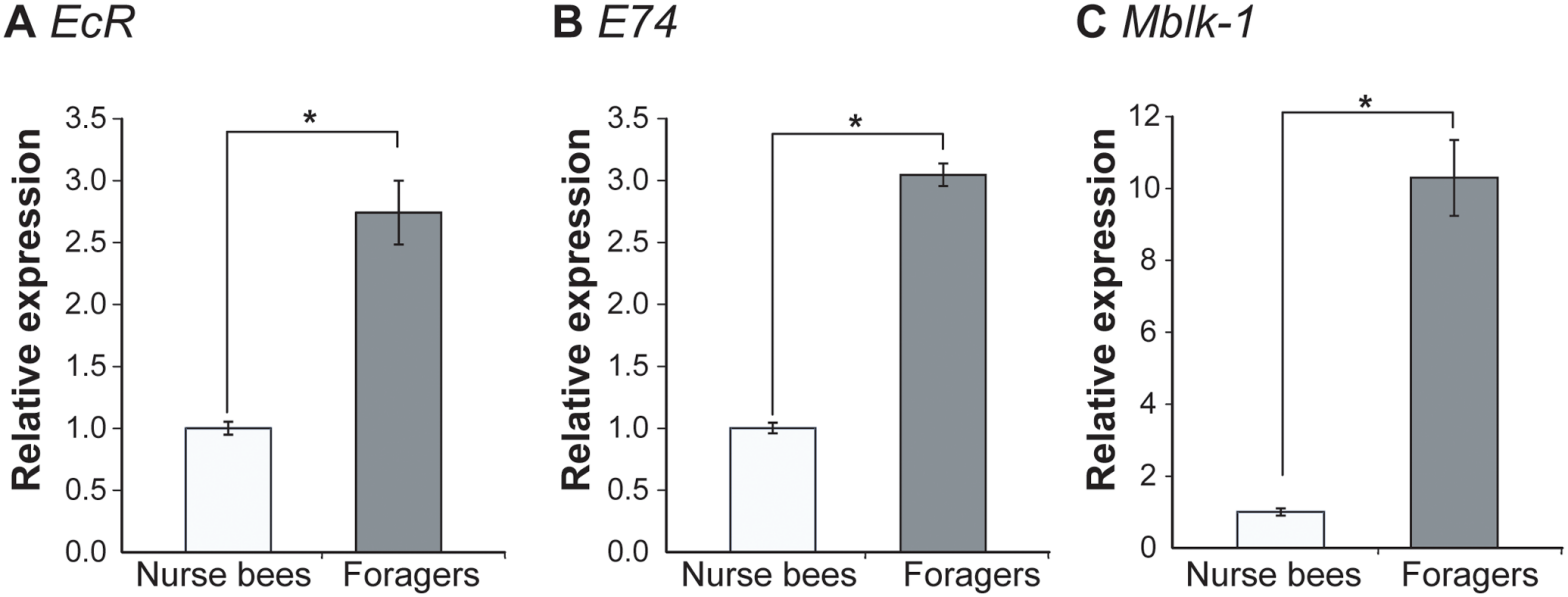
Quantification of ecdysone-related gene transcripts in the HPGs of nurse bees and foragers. Nurse bees or foragers (n = 9-14/group) were collected as one batch, and a total of four batches prepared from four different normal colonies were subjected to real-time RT-PCR. Relative mRNA levels of *EcR* (A), *E74* (B), and *Mblk-1* (C) are indicated with the standard error, with the amount of mRNA in nurse bee HPGs defined as 1. Transcript amounts were normalized with that of *elongation factor 1α-F2* (*EF1α-F2*). Asterisks indicate significant differences between nurse bees and foragers (*, p < 0.05; t-test).

Quantitative reverse transcription-polymerase chain reaction (RT-PCR) analysis revealed that the *EcR* and *E74* expression levels were approximately 3.0-fold higher in foragers than in nurse bees (Welch’s t-test, p<0.01 and Student’s t-test, p<0.000001), and *Mblk-1* expression was approximately 10-fold higher in foragers than in nurse bees (Welch’s t-test, p<0.01) (Fig 3).

**Fig 4:**
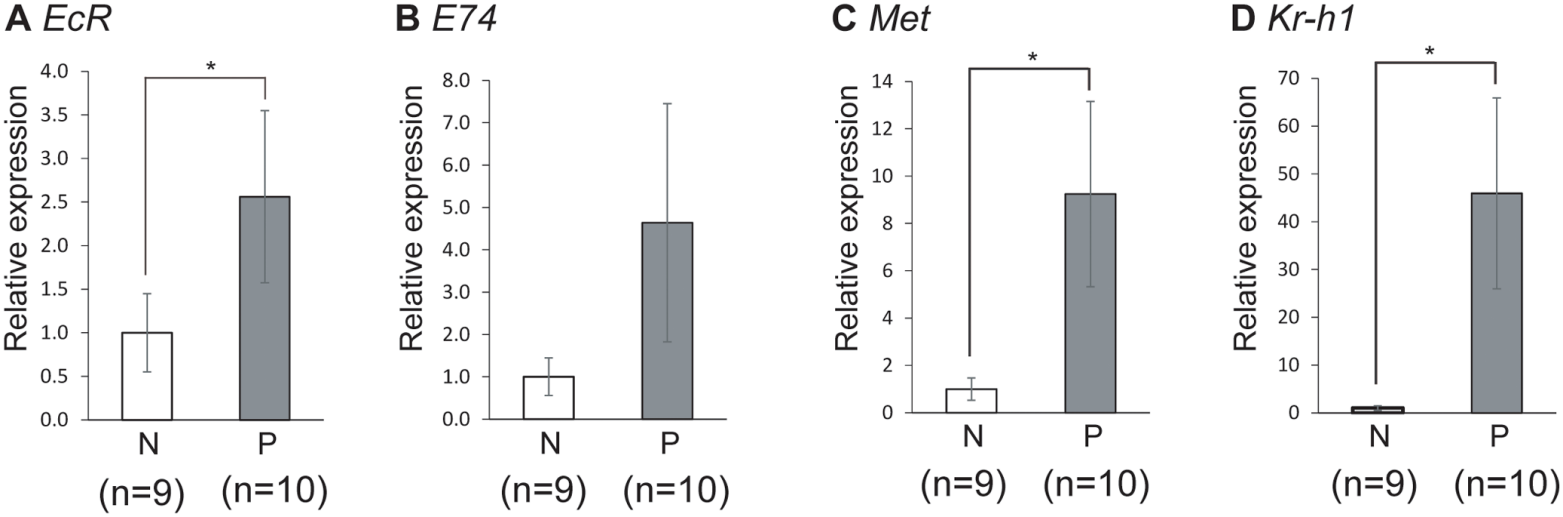
Quantification of ecdysone- and JH-related gene transcripts in the HPGs of nurse bees and precocious foragers derived from single-cohort colonies. Nurse bees (N) and precocious foragers (P) were collected from two single-cohort colonies. Relative mRNA levels of *EcR* (A), *E74* (B), *Met* (C), and *Kr-h1* (D) in the HPGs of workers derived from two single-cohort colonies are indicated with standard error. The amount of mRNA in the HPG of nurse bees is defined as 1. Transcript amounts were normalized with that of *ribosomal protein 49* (*rp49*). Asterisks indicate significant differences between nurse bees and precocious foragers (*, p < 0.05; t-test).

Subsequently, to examine whether the expression of ecdysone-related genes also correlates with workers’ role while controlling for age, we quantified the expression levels of *EcR*, *Mblk-1* and *E74* in the HPGs of nurse bees and precocious foragers derived from two single-cohort colonies. Comparison of mean expression levels in pooled samples from two colonies revealed that expression level of *EcR* was significantly (approximately 2.5-fold) higher in precocious foragers than in nurse bees as in normal colonies (Welch’s t-test, p<0.05), and *E74* expression was approximately 4.5-fold higher in precocious foragers than in nurse bees, although there was no significant differences (Welch’s t-test, p = 0.125) (Fig 4). We could not detect the expression of *Mblk-1* in the HPGs of most workers because the signal intensities were lower than the detection threshold (data not shown). Thus, we could not compare the *Mblk-1* expression between nurse bees and precocious foragers. Nevertheless, our findings indicate that the expression of ecdysone-regulated genes closely correlates with the workers’ role, as well as *mrjp2*, *Hbg3*, *Ambuffy* and *AmMMP1*. Considering that the expression levels of ecdysone-regulated genes are upregulated by ecdysone in *Drosophila* pupae [37, 39], these findings suggest that ecdysone signaling in the HPGs is activated in association with the role change of the worker honeybees from nursing to foraging.

### Expression analysis of genes in the HPGs of worker honeybees treated with 20-hydroxyecdysone (20E)

To more directly analyze the effect of ecdysone on gene expression levels in the HPGs, 20E solution was injected into the nurse bee heads, and quantitative analysis of gene expression in the HPGs was performed on days 1 and 3 after the treatment. In various insect species, including the honeybee, ecdysone catalyzed by cytochrome P450, CYP 314A1, which is 20E-monooxigenase encoded by the *shade* gene, is converted to the active form of ecdysone, 20E [35, 40].

We injected nurse bees in the present study because we hypothesized that ecdysone-signaling has a role in determining the gene expression profile in forager HPGs. On the other hand, we did not inject foragers because there are few reports that show reversal of task transition from foragers to nurse bees among worker honeybees. In addition, 20E was only injected into the bees once, as repeatedly anesthetizing the bees for injection of the 20E solution was technically difficult and thought to lead to high mortality. In our preliminary experiments, the gene expression profiles were examined at 3 and 7 days after 20E treatment. The gene expression levels, however, did not differ between 20E-treated and control bees at 7 days after the treatment (data not shown). Therefore, we planned to examine gene expression at 1 and 3 days after the treatment. Finally, we chose to inject 1 μl of 20E concentrated at 5 mM (2.5 μg/μl) based on the fact that the peak ecdysone titer is approximately 100 nM (50 ng/μl) during honeybee metamorphosis [41]. The amount of injected 20E was thought to be sufficient for analyzing the effect of 20E given that the amount was much higher than the hemolymph ecdysone titer.

**Fig 5:**
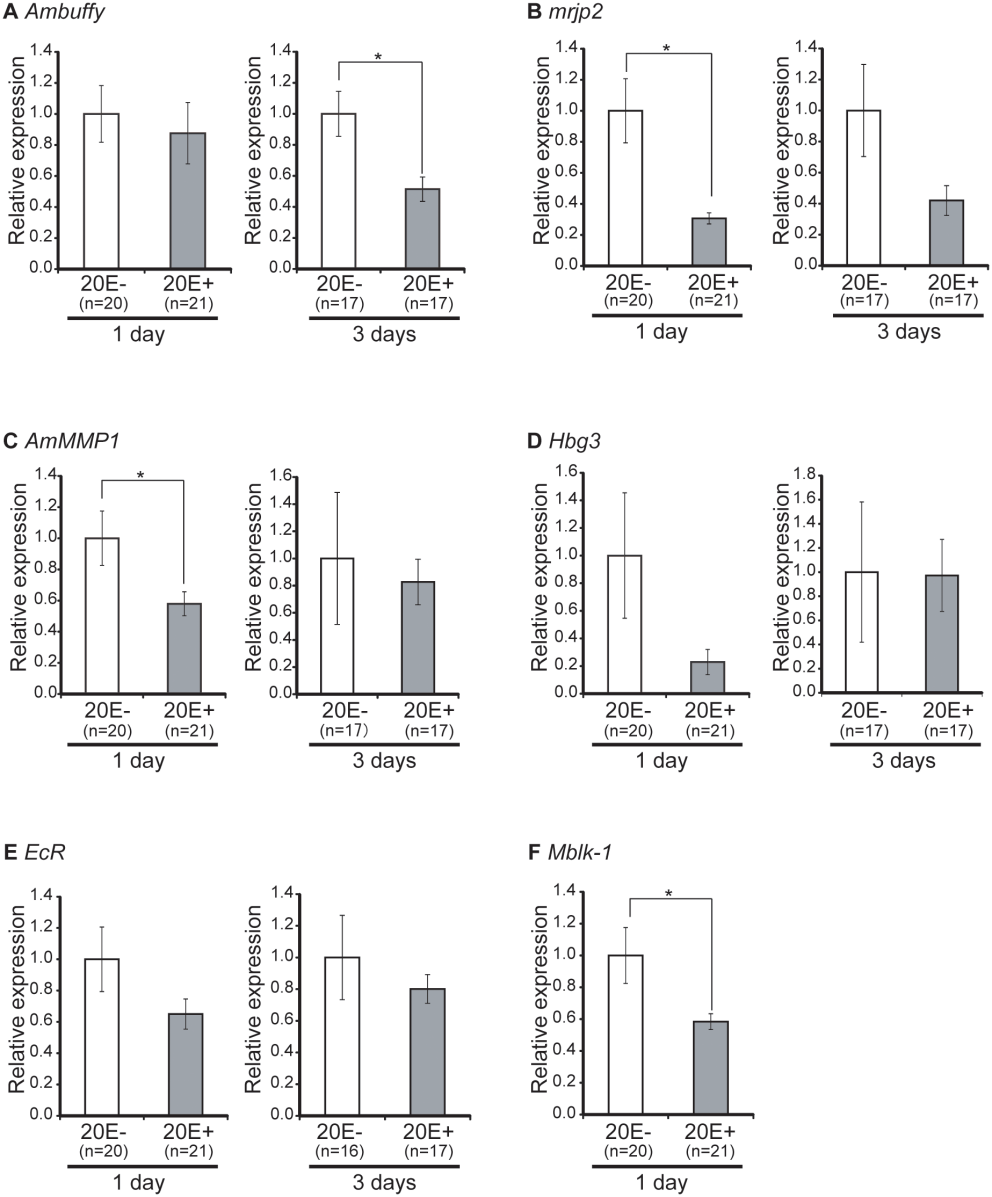
Quantification of gene expression in the HPGs of nurse bees treated with 20-hydroxyecdysone. Nurse bees that were actively feeding the brood were collected from normal colonies. The 20E solution (1μl; 2.5μg/μl) was injected in the anterior aspect of the head. HPGs were dissected out from worker heads and subjected to quantitative RT-PCR analysis at 1 and 3 days after treatment. A total of two or three trials were performed to confirm the reproducibility. Gene transcripts were quantified from pooled samples obtained from all trials. Relative mRNA levels of *Ambuffy* (A), *mrjp2* (B), *AmMMP1* (C), *Hbg3* (D), *EcR* (E), and *Mblk-1* (F) are indicated with the standard error, with the amount of mRNA in the HPGs of control bees defined as 1. Transcript amounts were normalized with that of *elongation factor 1α-F2* (*EF1α-F2*). Asterisks indicate significant differences between 20E-treated bees and control bees (*, p < 0.05; t-test).

On day 3 after 20E treatment, the mRNA level of *Ambuffy*, a nurse bee-selective gene, was significantly (~50%) lower in 20E-treated bees than in control bees (Welch’s t-test, p<0.01), while on day 1 after treatment, the *Ambuffy* mRNA level did not significantly differ between 20E-treated and control bees (Student’s t-test, p = 0.647) (Fig 5). The mRNA level of *mrjp2*, which is also a nurse bee-selective gene encoding a major royal jelly protein, was significantly (~70%) lower in 20E-treated bees than in control bees on day 1 after treatment (Welch’s t-test, p<0.01), and approximately 60% lower in 20E-treated bees than in control bees on day 3 after the treatment, although the latter decrease was not statistically significant (Welch’s t-test, p = 0.0786) (Fig 5). The changes in these gene expression levels appeared to mimic the changes in the expression levels of *Ambuffy* and *mrjp2*, both of which are nurse bee-selective genes whose expression decreases in association with the role change of the workers. There are some possible explanations for the differential effects of 20E on the expression of *Ambuffy* and *mrjp2*. For example, it might be that 20E temporarily represses *mrjp2* expression and the resulting decline in *mrjp2* expression leads to a decrease in *Ambuffy* expression while *mrjp2* expression resumes. Another possibility is that repression of *Ambuffy* expression requires developmentally regulated factor(s) other than 20E, such as JH whose titer in the hemolymph increases over the worker’s lifetime, because gene expression was analyzed 3 days after 20E treatment and as the bees got older.

The mRNA levels of *AmMMP1* and *Hbg3*, which are forager-selective genes, did not differ significantly between 20E-treated and control bees on day 3 after the treatment (Welch’s t-test, p = 0.740 and p = 0.967, respectively), whereas on day 1 after treatment, mRNA levels of *AmMMP1* were significantly (~40%) lower in 20E-treated bees than in control bees (Welch’s t-test, p<0.05), and mRNA levels of *Hbg3* were approximately 80% lower in 20E-treated bees than in control bees, although the latter decrease was not statistically significant (Welch’s t-test, p = 0.111) (Fig 5). These findings suggest that ecdysone is not involved in inducing the expression of *AmMMP1* and *Hbg3*.

Quantification of the transcripts for *EcR* and *Mblk-1*, which are ecdysone-regulated genes and whose expression in the HPGs increases with role change of workers, revealed that the mRNA levels of *EcR* in the HPGs were approximately 20% to 40% lower in 20E-treated bees than in control bees on days 1 and 3 after treatment (Fig 5), although the decreases were not statistically significant (Welch’s t-test, p = 0.136 and p = 0.489, respectively). The *Mblk-1* mRNA level in the HPGs was significantly (~40%) lower in 20E-treated bees than in control bees on day 1 after the treatment (Welch’s t-test, p<0.05) (Fig 5). The *Mblk-1* expression level on day 3 after treatment was not examined. Although *EcR* and *Mblk-1* are reported to be upregulated in response to increased ecdysone levels in *Drosophila* pupae [42, 43], these findings suggest that the expression of *EcR* and *Mblk-1* is regulated rather by negative feedback, and expression of these genes in forager HPGs could be induced by other internal factors.

### Expression analysis of genes related to JH signaling in the HPGs of workers associated with the role change

We then examined the possible involvement of JH signaling in regulating HPG physiology in the worker honeybees. *Methoprene tolerant* (*Met*) and *Krüppel-homolog 1* (*Kr-h1*) are thought to be JH signaling-related genes [44–50]. Met is a putative JH receptor and mediates JH action in almost all insects. The expression level of *Met* mRNA is important for predicting the JH action: in *Tribolium castaneum*, the *Met* mRNA level in the whole body increases at the end of the larval stage, suggesting that Met mediates JH action at this stage [48]. On the other hand, JH or methoprene application induces the expression of *Kr-h1* in *T*. *castaneum* pupae, indicating that *Kr-h1* is a JH-response gene [46]. To investigate whether the function of JH signaling in the HPGs changes in association with the role change of the worker honeybees, we quantified the amount of transcripts of *Met* and *Kr-h1* in the HPGs of nurse bees and foragers derived from normal colonies. For this, 9 to 14 nurse bees and foragers were collected at the same time from a single colony, and gene expression was compared using a total of four batches of samples derived from four different colonies.

**Fig 6:**
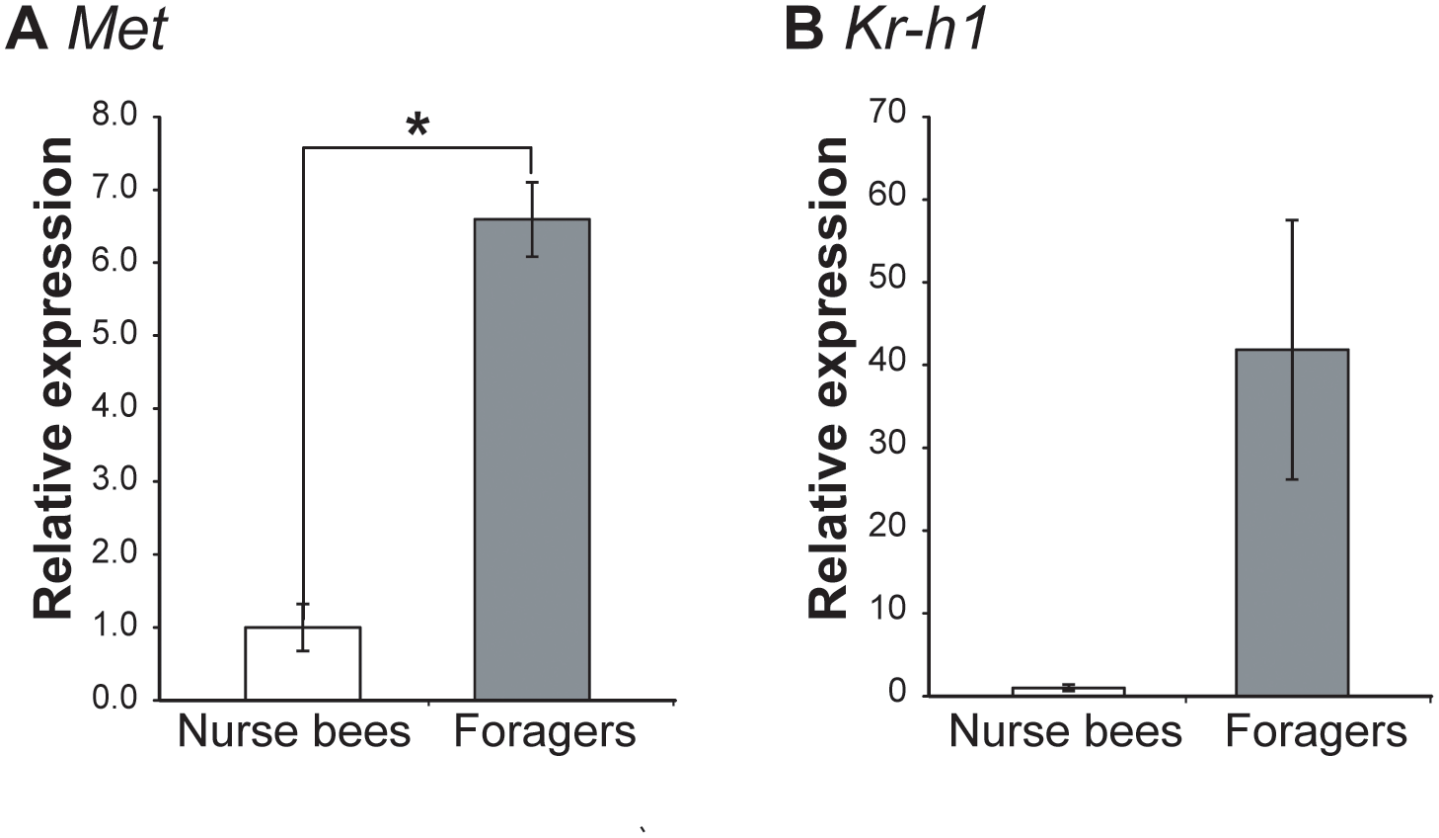
Quantification of JH-related gene transcripts in the HPGs of nurse bees and foragers. Nurse bees or foragers (n = 9-14/group) were collected as one batch, and a total of four batches prepared from four different colonies were subjected to quantitative RT-PCR. Relative mRNA levels of *Methoprene tolerant* (A) and *Krüppel homolog 1* (B) are indicated with standard error, with the amount of mRNA in nurse bee HPGs defined as 1. Transcript amounts were normalized with that of *elongation factor 1α-F2* (*EF1α-F2*). Asterisks indicate significant differences between nurse bees and foragers (*, p < 0.05; t-test).

Quantitative RT-PCR analysis revealed that the *Met* expression level in the HPGs was significantly (~6.5-fold) higher in foragers than in nurse bees (Student’s t-test, p<0.0001), and the *Kr-h1* expression level tended to be approximately 40-fold higher in foragers than in nurse bees, although the increase in *Kr-h1* expression was not statistically significant (Welch’s t-test, p = 0.121) (Fig 6).

We also compared the expression levels of *Met* and *Kr-h1* in the HPGs between nurse bees and precocious foragers derived from two single-cohort colonies. Comparison of mean expression levels in pooled samples from two colonies revealed that expression levels of *Met* and *Kr-h1* were significantly (approximately 9.0-fold and 45-fold, respectively) higher in precocious foragers than in nurse bees, as in normal colonies (Welch’s t-test, p<0.05 and p<0.001, respectively) (Fig 4). These findings suggest that JH signaling in the HPGs increases in association with the role change of the worker honeybee, consistent with the previous report that JH stimulates physiological changes in the HPGs in terms of gland size and the enzymatic activity of α-glucosidase [13].

### Expression analysis of genes in the HPGs of worker honeybees treated with methoprene

With the aim to examine the effect of JH analogue on gene expression in the HPGs, we applied methoprene to 6-day old worker heads, and analyzed gene expression in the HPGs on day 7 after treatment.

**Fig 7:**
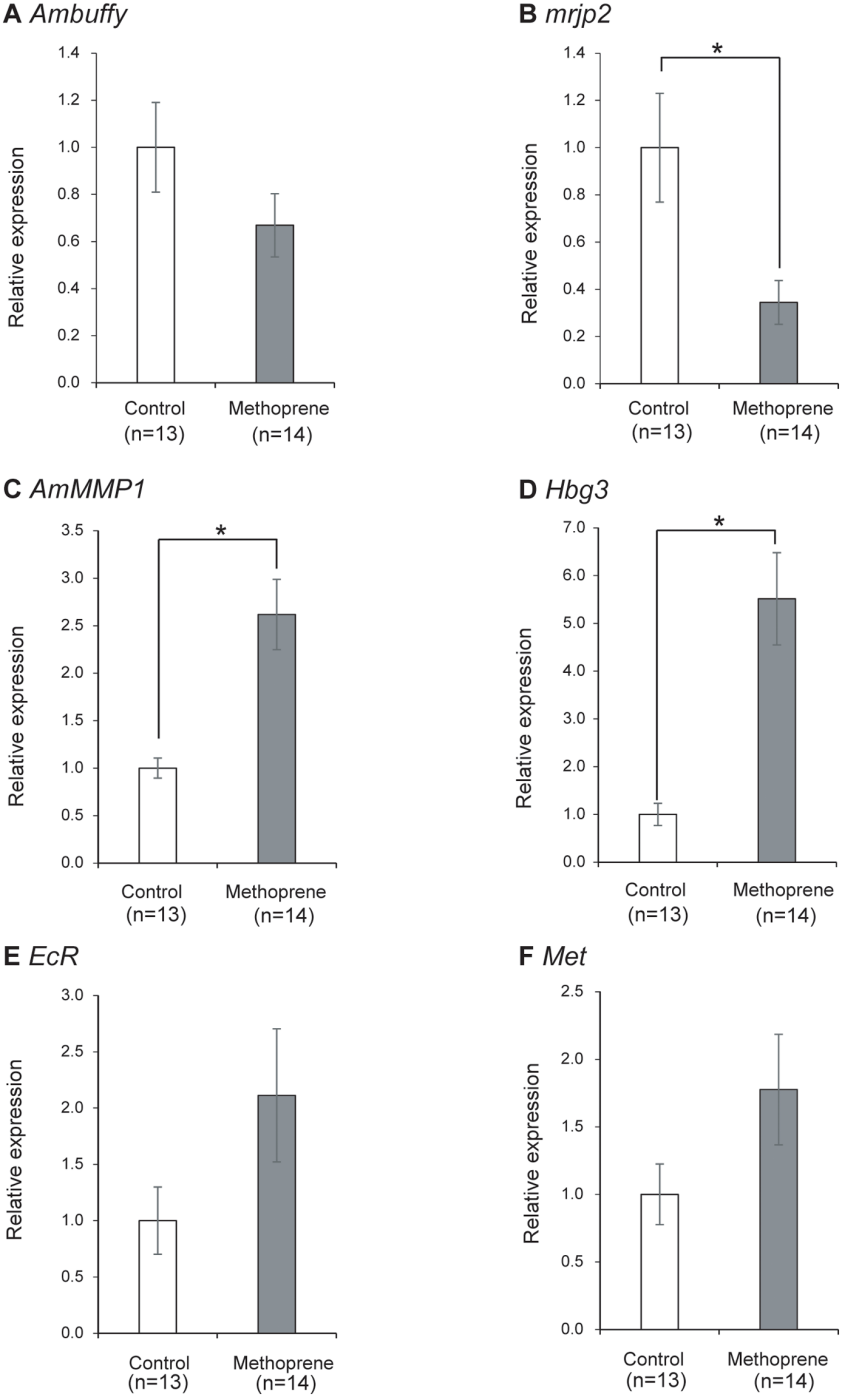
Quantification of gene expression in the HPGs of worker honeybees treated with methoprene. Methoprene (250 μg) dissolved in acetone was applied to the heads of 6-day old workers. HPGs were dissected out from worker heads and subjected to quantitative RT-PCR analysis at 7 days after treatment. A total of two trials were performed to confirm the reproducibility. Gene transcripts were quantified from pooled samples obtained from all trials. Relative mRNA levels of *Ambuffy* (A), *mrjp2* (B), *AmMMP1* (C), *Hbg3* (D), *EcR* (E), and *Met* (F) are indicated with the standard error, with the amount of mRNA in the HPGs of control bees defined as 1. Transcript amounts were normalized with that of *elongation factor 1α-F2* (*EF1α-F2*). Asterisks indicate significant differences between methoprene-treated bees and control bees (*, p < 0.05; t-test).

Quantitative RT-PCR analysis revealed that the mRNA level of *Ambuffy*, a nurse bee-selective gene, was approximately 30% lower in methoprene-treated bees than in control bees (Student’s t-test, p = 0.173), while the mRNA level of *mrjp2*, which was also a nurse bee-selective gene, was significantly (~65%) lower in methoprene-treated bees than in control bees (Welch’s t-test, p<0.05) (Fig 7). On the other hand, the mRNA levels of *AmMMP1* and *Hbg3*, which were forager-selective genes, were significantly higher (2.5 to 5.5-fold) in the methoprene-treated bees than in the control bees (Welch’s t-test, p<0.001 and p<0.001, respectively) (Fig 7). The changes in these gene expression levels were similar to the changes of the gene expression levels in HPGs in association with the role change from nursing to foraging in normal colonies. In addition, we examined the gene transcripts of *EcR* and *Met*, which are ecdysone- and JH-related genes, respectively. The results indicated that the mRNA levels of these two genes were approximately 2.0-fold higher in methoprene-treated bees than in control bees, as well as the mRNA levels of *AmMMP1* and *Hbg3*, although the decreases were not statistically significant (Welch’s t-test, p = 0.111 and Student’s t-test, p = 0.103) (Fig 7). These findings suggest that JH downregulates expression levels of nurse bee-selective genes, whereas upregulates the expression levels of forager-selective genes in the HPGs. Finally, it should be emphasized that we did not examine the effect of hormone-treatment on worker behavior in the present study because hormone-injected workers were expelled from their colonies by the other workers. Therefore, our experiments only confirmed gene expression changes and did not address causation of the worker behavior.

## Discussion

Gene expression analysis using single-cohort colonies in which workers of almost the same age performed different tasks indicated that the expression of not only *mrjp2* and *Hbg3*, but also *Ambuffy* and *AmMMP1*, correlated with worker behavior while controlling for age, even in single-cohort colonies. Many studies from several laboratories using single-cohort colonies have demonstrated that expression of some genes in the brain changes in association with the behavior of the workers [51–53], suggesting that brain function changes depending on the task transition. Further, based on analysis of workers from single-cohort colonies, Mutti et al. (2011) found that gene expression not only in the brain but also in the fat bodies and ovaries correlates with worker behavior [54]. In the present study, the physiological state of the HPGs (expression of these ‘indicator’ genes) changed in association with worker behavior, suggesting the concerted regulation of gene expression profiles in the HPGs. Moreover, our findings imply that changes in the expression levels of these ‘indicator’ genes in response to worker behavior are cooperatively regulated by some internal factors.

The candidate internal factors, which cooperatively regulate HPG physiology and worker behavior, include ecdysone and JH. In *Drosophila melanogaster*, *Dmbuffy* and *DmMMP1* expression are regulated by ecdysone signaling in cell death during metamorphosis [37, 39]. Thus, it is plausible that *Ambuffy* and *AmMMP1* expression is also regulated by ecdysone in the worker HPGs. In addition, our previous studies revealed that some ecdysone signaling-related genes, *EcR*, *E74*, and the gene for a novel transcription factor, *mushroom body large type Kenyon cell specific protein-1* (*Mblk-1*), as well as *Nuclear hormone-like 38*, which encodes a hormone receptor resembling but distinct from *EcR*, are expressed in the brains of worker honeybees [31–34], suggesting the possible involvement of ecdysone signaling in regulating adult honeybee brain function. Based on these findings from previous studies and our present study, we speculated that ecdysone regulates brain function and HPG function cooperatively in response to the task transition of workers.

Subsequently, with the aim to analyze the involvement of ecdysone signaling in the coordinated regulation of the physiological state of HPG and worker behavior, we compared the expression levels of the ecdysone-response genes *EcR*, *Mblk-1*, and *E74* in the HPGs between nurse bees and foragers in normal colonies, and between nurse bees and precocious foragers derived from single-cohort colonies. The results suggest that ecdysone signaling in the HPGs is activated in association with the task transition of worker honeybees from nursing to foraging. In addition, we analyzed the change in the expression of nurse bee- and forager-selective genes as well as those of ecdysone-related genes in the HPGs of ecdysone-treated nurse bees. We found that 20E repressed the expression of *Ambuffy* and *mrjp2*, which are nurse bee-selective genes in the HPGs, raising the possibility that ecdysone represses the nurse bee-type physiological state of the HPGs in association with the task transition of workers from nursing to foraging. The expression of *AmMMP1* and *Hbg3*, both of which are forager-selective genes, however, is also repressed by 20E injection, suggesting that other endocrine factors are involved in the induction of the forager-bee type HPG physiological state. In addition to these genes, injection of 20E into the honeybee head repressed the expression of *EcR* and *Mblk-1* in the HPGs. Mello et al. indicated that application of 20E (5.0 μg) reduces the expression of *EcR* in the fat bodies of pharate adult honeybees, suggesting that a high concentration of 20E represses *EcR* expression [55]. Additionally, Velarde et al. reported that the expression of some ecdysone-response genes in the brain is repressed by injection of 20E (5.0 μg) [56]. Our results are consistent with these previous studies. Taken together, a high concentration of 20E might repress some ecdysone-regulated genes, including *EcR* and *Mblk-1/E93*, in the honeybee. Therefore, it might be that expression of *EcR* and *Mblk-1* in HPGs treated with 20E reflects a negative feedback effect of 20E, because we injected an aliquot of the high dose (2.5 μg/μl) of 20E.

In addition to ecdysone signaling, we examined the possible involvement of JH signaling in regulating HPG physiology. In some insect species, such as the fruit fly *Drosophila melanogaster* and the beetle *Tribolium castaneum*, Met and Kr-h1 are key components in JH signaling. Met is thought to be a candidate JH receptor, because Met, which is a transcription factor of the basic helix-loop-helix Per-Arnt-Sim family, was originally identified in mutant flies resistant to a JH mimic, methoprene [44, 50]. Furthermore, *Drosophila* Met protein binds to JH-III with high affinity [47]. In *T*. *castaneum*, Met knockdown causes a precocious larval-pupal transition, and suppresses responsiveness to an exogenous JH mimic [45, 48]. On the other hand, in *T*. *castaneum*, Kr-h1, which is a transcription factor with homology in the zinc (Zn)-finger motifs and amino acid spacer connecting the Zn-finger motifs [49], is an early JH-response gene that mediates JH action downstream of Met [46].

In our present study, quantitative analysis of *Met* and *Kr-h1* expression in the HPGs suggested that JH signaling is activated in association with task transition from nursing to foraging. To analyze the direct relationship between JH and gene expression in the HPGs, we performed two trials to quantitatively analyze gene expression in the HPGs of 6-day old workers treated with methoprene. The results indicated that methoprene induced the expression of forager-selective genes (*AmMMP1*, *Hbg3*, *EcR* and *Met*) whereas expression levels of nurse bee-selective genes (*Ambuffy* and *mrjp2*) were decreased in methoprene-treated bees. In adult worker honeybees, the JH titer in the hemolymph increases with behavioral development, and an elevated JH titer increases the enzymatic activity of α-glucosidase in the HPGs and contracts the gland tissues [13]. Thus, the expression of forager-selective genes seems to be induced according to an increase in the JH titer in hemolymph, and the effect of JH on these gene expression in the HPGs might be more prominent than that of ecdysone, or the effective period for ecdysone and JH might be temporally segregated in association with the division of labor of workers. Although the effect of JH on the expression of *mrjps* in the HPGs had not been examined, JH was believed to downregulate them in the HPGs [57]. In the present study, we revealed that JH application downregulates expression levels of nurse bee-selective genes (*mrjp2* and *buffy*) in the HPGs, supporting the hypothesis that JH reprograms the HPG function [57].

In contrast to JH, ecdysone was not considered to relate to the division of labor and physiology of the HPGs, because the ecdysone titer in the hemolymph of workers does not change throughout life after eclosion (~2 to 6 pg/μl) [38, 41, 58, 59]. On the other hand, JH not only accelerates task transition, but also changes the physiology of the HPGs [13]. JH signaling alone, however, does not provide an adequate explanation for the establishment of the age-related division of labor, because JH is not necessary for behavioral development but accelerates task transition, which is why JH is called a 'pacemaker' [21]. Yamazaki et al. suggested that ecdysone is synthesized in the brains, fat bodies, and HPGs, and proposed that the switch of ecdysone signaling in the brain is related to the task transition [34, 35]. Our findings further suggest that ecdysone synthesized in the HPGs might directly regulate the transcription of *mrjp2* and *Ambuffy*, thereby coordinating the physiological state of HPG with the worker behavior. Thus our findings first revealed a role of ecdysone in transition of the physiological state of HPGs from nurse bee to forager. It is assumed that ecdysone and JH act cooperatively to alteration of gene expression in the HPGs in association with the role change of workers, although the effect of ecdysone on the gene expression is more restricted than that of JH. JH is known to regulate the physiological state of individuals associated with task specialization in social hymenopteran insects, as well as in the honeybee [60, 61], while there are only a few reports of the involvement of ecdysone. A better understanding of the cooperative regulatory mechanism underlying the physiological state of the HPGs by both ecdysone and JH will elucidate the molecular foundation of the division of labor in social insects. Many studies in honeybees have supported the reproductive ground plan hypothesis, which proposes that reproductive traits are linked to task specialization of workers in social insects. Wang et al. (2012) suggested that ecdysone and JH may be involved in both brain and ovary physiologies related to foraging behavior [30]. Perhaps these two hormones, both of which play a gonadotropic role in insect reproduction, might regulate the physiologies of peripheral organs and behavioral development of worker honeybees in association with role change. Currently, nutritional state (titer of vitellogenin) is believed to act on the regulation of behavioral development via insulin/insulin-like signaling (IIS) pathway which acts upstream of JH synthesis [10]. In this model, the fat body physiology is closely related to worker behavior; the expression of insulin-like peptides in the fat body influences brain function according to the nutritional state. Additionally, an increase in the ovarian mass induces behavioral development [28], and gene expression profiles in the ovary correlate with those in the brain in response to socio-environmental factors [30], suggesting that ovary physiology is also related to worker behavior. It remains to be determined, however, whether the HPGs influence worker behavior like the fat bodies and ovaries. The HPG, which synthesizes and secretes high amount of proteinaceous materials for feeding the brood, serves a storage function (storage of major royal jelly proteins) in winter bees [62]. Therefore, the HPGs might play a crucial role in the worker’s nutritional state, which regulates behavioral development. Further analysis of the regulatory mechanisms of HPG activity and the relationship with trophic conditions will help to clarify the role of HPGs in behavioral development.

## Materials and Methods

### Animals and tissues

European honeybee (*Apis mellifera* L.) colonies were purchased from the Kumagaya bee farm (Saitama, Japan) and maintained at the University of Tokyo (Tokyo, Japan). Nurse bees were collected when they were actively feeding the brood and their HPGs were well developed; forager bees were collected when they returned to the colony after foraging pollen and honey, and their HPGs were shrunken [12]. After the workers were anesthetized on ice, the heads were removed from the bodies, and the HPGs were dissected from the heads with fine tweezers and a surgical knife under a binocular microscope. For RNA extraction, the HPGs were stored frozen at -80°C until use.

### Preparation of single-cohort colonies

From three normal colonies, several combs that contained pupae were collected. The combs that contained pupae were distinguishable from other combs, as these combs were sealed. After all adherent bees were removed, the collected combs were incubated at 33°C in an incubator. Approximately 6000 newly emerged workers were collected for 3 days, and paint marks were applied to the thorax of approximately 900 workers using poster paint, POSCA (Mitsubishi Pencil, Co., Ltd. Tokyo, Japan) to ensure that the sampled workers (only the marked workers were collected in subsequent experiments) were derived from the single-cohort colonies. The quantity of workers introduced to each colony was determined by the weight of five workers randomly collected from normal colonies. Two single-cohort colonies (colony Nos. 1 and 2), each of which comprised a single queen and approximately 3000 workers, were created by introducing the queen and the newly-emerged marked workers. Each colony was given one comb with honey and pollen as preserved foods, and one empty comb for egg-laying by the queen. Six to eight days after creating the single-cohort colonies, nurse bees that were taking care of their brood by secreting royal jelly and precocious foragers that returned to their hives with a pollen load, both of which had paint-marks on the thorax, were collected as described above [12].

### 20-hydroxyecdysone (20E) treatment

20-hydroxyecdysone (Sigma-Aldrich, St. Louis, MO) was diluted in one part of ethanol and three parts insect saline (130 mM NaCl, 5 mM KCl, 1mM CaCl_2_) to a concentration of 2.5 μg 20E/μl. Nurse bees that were actively feeding the brood were collected from normal colonies. The HPG morphology was not examined for convenience of the experiment. Collected nurse bees were anesthetized at 4°C in a refrigerated chamber and kept on ice until solutions were injected. The anesthetized bees were immobilized on dental wax using tweezers. One microliter of the 20E solution was injected into the anterior aspect of the head. The injection tip (Drummond Scientific Company, Broomall, PA) was inserted through the base of the antennae. Control experiments were performed using solvent. Honeybees of each group were reared in cages in an incubator under dark conditions at 33°C for 1 or 3 days. The supplied diet comprised 50% honey and 50% water (v/v). After 1 or 3 days rearing, surviving honeybees of each group were anesthetized in a 4°C refrigerated chamber and the HPGs were dissected out from the heads.

### Methoprene treatment

Newly emerged worker honeybees collected from normal colonies were marked with poster paint on their thorax, and returned to their colonies. After 6 days, painted workers (6-day old) were collected from colonies. After anesthetizing at 4°C, 250 μg of methoprene (Sigma-Aldrich), which was dissolved in acetone, was applied to their heads. Control experiments were performed using solvent. Honeybees of each group were reared in cages in an incubator under dark conditions at 37°C for 7 days. The supplied diet comprised 50% honey and 50% water (v/v). After 7 days rearing, surviving honeybees of each group were anesthetized at 4°C and the HPGs were dissected out from the heads.

### Quantitative RT-PCR

Total RNA was extracted from the HPGs, which were dissected from worker honeybees, using TRIZol Reagent (Invitrogen, Carlsbad, CA). Total RNA was then treated with DNase I and reverse-transcribed using SuperScript III (Invitrogen) or PrimeScript RT reagent kit (TAKARA BIO Inc., Ohtsu, Japan). Real-time PCR was performed with SYBR premix Ex Taq II (TAKARA BIO Inc.) or LumminoCt SYBR Green qPCR Ready mix (Sigma-Aldrich), using LightCycler (F. Hoffmann-La Roche, Ltd. Basel, Switzerland) or Eco Real-time PCR system (Illumina Inc. San Diego, CA) according to the manufacturer’s protocol, using gene-specific primers. The gene specific primers (*Ambuffy*; 5’-CATGGCACTTCTCATCCTTTTC-3’ and 5’-GAGAACGGTTTCAGCATCAATC-3’, *AmMMP1*; 5’-GCTTCCCGATAATCTTGATG-3’ and 5’-CATCCGAACCACCAGTAAG-3’, *mrjp2*; 5’-AAATGGTCGCTCAAAATGACAGA-3’, and 5’-ATTCATCCTTTACAGGTTTGTTGC-3’, *Hbg3*; 5’-TACCTGGCTTCGTGTCAAC-3’ and 5’-ATCTTCGGTTTCCCTAGAGAATG-3’, *EcR*; 5’-GAGGTGATGATGCTTCGAATG-3’ and 5’-CCGGCAGAAATGTAGCAAATC-3’, *E74*; 5’-CCGAAAGCTACAGCAGTTATG-3’ and 5’-CCAGTAGATATAAATCGTCGGAAAC-3’, *Mblk-1*; 5’-CAACACCAAATACGACCCAAAAC-3’ and 5’-GACAACAGCGGCTTCAAC-3’, *Met*; 5’- CAACATTTACCTCCTGCTGAAG-3’ and 5’- GATCTCGTGTTTTCTTGTCTCTC-3’, *Kr-h1*; 5’- TTGGAAGCAGTTGAAGAAGAAAG-3’ and 5’- CGTACAGGAATCGCCAAATC-3’) were derived from cDNA sequences for each gene and information from the Honey Bee Genome Resource (S1 File). Primers for *EcR* amplification were designed based on the nucleotide sequences corresponding to the common region of both *EcRA* and *EcRB1* isoforms (Fig A in S1 File). Thus, quantitative RT-PCR analysis of *EcR* was expected to detect the total amounts of *EcRA* and *EcRB1* transcripts. *Drosophila melanogaster*, *Bombyx mori*, and *Aedes aegypti* have two isoforms of *E74*, *E74A*, and *E74B* [63–65]. Although the cDNA sequence corresponding to the *E74A* isoform has been isolated in the honeybee [32], the cDNA sequence corresponding to the *E74B* isoform has not yet been isolated. Therefore, primers for quantitative RT-PCR of *E74* were designed based on the nucleotide sequence of a part of the *E74A* cDNA (Fig B in S1 File). PCR conditions were: [95°C × 30 s + (95°C × 5 s + 60°C × 15 s + 72°C × 20 s) × 45–55 cycles] or [95°C × 20 s + (95°C × 5 s + 60°C × 20–60 s) × 45 cycles]. Transcript amounts were normalized with that of *elongation factor 1α-F2* (*EF1α-F2*) [66] or *ribosomal protein 49* (*rp49*) [67]. There were no significant differences in the expression levels of *EF-1α-F2* and *rp49* between the HPGs of nurse bees and foragers (data not shown).

### Statistical analysis

Statistical analysis was performed using Statcel3 (The publisher OMS Ltd., Saitama, Japan). The amounts of gene transcripts were compared between two experimental groups (nurse bees vs. foragers, nurse bees vs. precocious foragers, 20E-treated bees vs. control bees, and methoprene-treated bees vs. control bees) using a two-tailed Student’s t-test. If F-test did not assume the homogeneity of variance, a two-tailed Welch’s t-test was used instead of Student’s t-test.

## Supporting Information

S1 File Genomic organization of ecdysone- and JH-related genes. Genomic organization of the genes for *EcR*(A), *E74*(B), *Mblk-1* (C), *Met* (D), and *Kr-h1* (E). Exon (filled boxes) and intron (lines) structure of each gene is indicated below the corresponding linkage group. Because the full-length cDNA sequence for the honeybee *Met* has not yet been isolated, putative cDNA sequences predicted by NCBI Honey Bee Genome Resources were used to speculate the genomic organization of the honeybee *Met*. Arrowheads indicate the positions of primers designed to amplify each transcript.
(TIF)
