# Supplementary figures and images for: Changes in the Gene Expression Profiles of the Hypopharyngeal Gland of Worker Honeybees in Association with Worker Behavior and Hormonal Factors
Source: PLoS One. 2015 Jun 17;10(6):e0130206. doi: 10.1371/journal.pone.0130206 (PMC4470657; doi:10.1371/journal.pone.0130206)

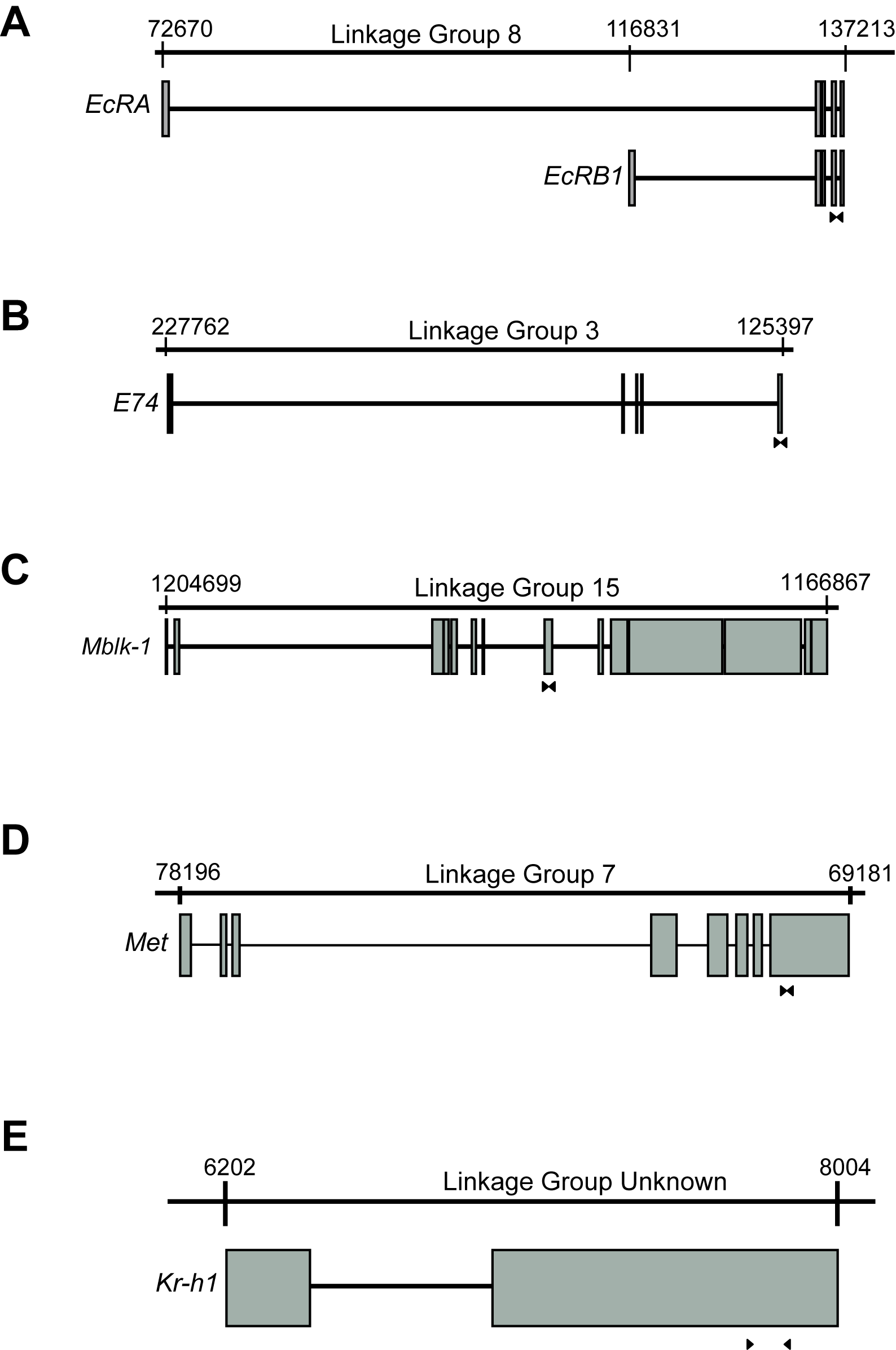

Supplement: S1 File — Genomic organization of the genes for EcR(A), E74(B), Mblk-1 (C), Met (D), and Kr-h1 (E). Exon (filled boxes) and intron (lines) structure of each gene is indicated below the corresponding linkage group. Because the full-length cDNA sequence for the honeybee Met has not yet been isolated, putative cDNA sequences predicted by NCBI Honey Bee Genome Resources were used to speculate the genomic organization of the honeybee Met. Arrowheads indicate the positions of primers designed to amplify each transcript. (TIF) [file pone.0130206.s001.tif]
